# Employing aromatic tuning to modulate output from two-component signaling circuits

**DOI:** 10.1186/s13036-015-0003-2

**Published:** 2015-05-16

**Authors:** Rahmi Yusuf, Roger R Draheim

**Affiliations:** Division of Pharmacy, Durham University, Queen’s Campus, Stockton-on-Tees, TS17 6BH England UK; School of Pharmacy and Biomedical Sciences, University of Portsmouth, St. Michael’s Building, White Swan Road, Portsmouth, PO1 2DT, England UK

**Keywords:** Synthetic microbiology, Aromatic tuning, Receptor engineering, Signal modulation, Synthetic circuits

## Abstract

Two-component signaling circuits (TCSs) govern the majority of environmental, pathogenic and industrial processes undertaken by bacteria. Therefore, controlling signal output from these circuits in a stimulus-independent manner is of central importance to synthetic microbiologists. Aromatic tuning, or repositioning the aromatic residues commonly found at the cytoplasmic end of the final TM helix has been shown to modulate signal output from the aspartate chemoreceptor (Tar) and the major osmosensor (EnvZ) of *Escherichia coli*. Aromatic residues are found in a similar location within other bacterial membrane-spanning receptors, suggesting that aromatic tuning could be harnessed for a wide-range of applications. Here, a brief synopsis of the data underpinning aromatic tuning, the initial successes with the method and the inherent advantages over those previously employed for modulating TCS signal output are presented.

## Introduction

Two-component signaling circuits (TCSs) are a ubiquitous mechanism by which bacteria sense, respond and adapt to external stimuli. TCSs facilitate responses to a wide range of environmental parameters, such as ambient temperature, availability of nutrients or external osmolarity [1]. Medically relevant multiorganism phenomena including quorum-sensing [2] and host-pathogen interaction are also governed by TCSs [3]. Furthermore, these systems control essential agricultural and environmental processes such as chloroplast synthesis [4] and nitrogen fixation [5], which is involved in root nodule formation. More than 400,000 open reading frames (ORFs) believed to encode TCSs have been sequenced and annotated suggesting that these systems control an almost unlimited number of processes that could be harnessed by synthetic microbiologists [6, 7]. Based on this premise, engineering TCSs with novel functionality is a very active field [8], especially because it has been recently shown that these modified systems can be systematically transferred to mammalian cells [9]. However, rationally designing TCSs, either by moving intact components from one microorganism to another or by forming chimeric components from the domains of two different proteins, can result in aberrant signal output or loss of stimulus-perception, or both [10]. Here, an aromatic tuning methodology that has been established to circumvent these issues and is generally applicable to both native and rationally designed TCSs is described.

### Two-component signaling circuits

**Fig. 1:**
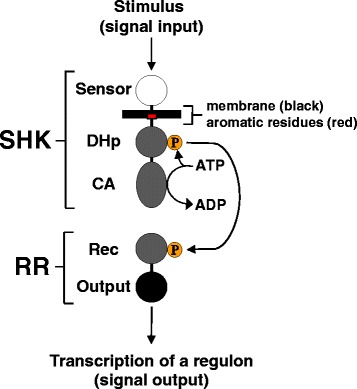
Modularity of two-component signaling circuits (TCSs). When the sensor domain of a canonical SHK perceives stimulus, communication occurs across the membrane (black line) resulting in increased kinase activity of the catalytic ATPase (CA) domain. This enhances phosphorylation of the conserved histidyl residue within the domain responsible for dimerization and histidylphosphotransfer (DHp). These nascent phosphoryl groups are subsequently transferred to an aspartyl residue within the receiver domain of the RR, which usually increases the DNA-binding activity of the output domain leading to transcription of a group of genes, known as a regulon, related to the cognate stimulus [1]. Aromatic tuning, or moving aromatic residues (red box) at the cytoplasmic end of the transmembrane (TM) domain, facilitates stimulus-independent modulation of signaling circuits by mimicking the presence of cognate stimulus and thus altering SHK output, and in turn, transcription of the associated regulon

Traditionally, a TCS consists of a membrane-spanning sensor histidine kinase (SHK) and a cytoplasmic response regulator (RR) [1], however, various higher-order protein architectures have been identified [11]. The largest group of membrane-spanning SHKs possesses a periplasmic or extracellular domain responsible for stimulus perception with signal transduction to the cell interior occurring via the adjacent transmembrane (TM) domain [12]. The extent of input stimulus usually controls the ratio of phosphorylation (kinase activity) to dephosphorylation (phosphatase activity) of the cognate RR, thereby regulating the intracellular level of phosphorylated RR [13]. Phosphorylation modulates the activity of the output domain, which normally interacts with DNA to control transcription of genes appropriate for mediating a response to the perceived stimulus (Fig. 1) [1]. Aromatic tuning, depicted as a red box at the cytoplasmic end of the TM domain in Fig. 1, facilitates stimulus-independent modulation of signaling circuits by mimicking the presence of cognate stimulus and thus altering SHK output.

### Biophysical and biochemical underpinnings of aromatic tuning

**Fig. 2:**
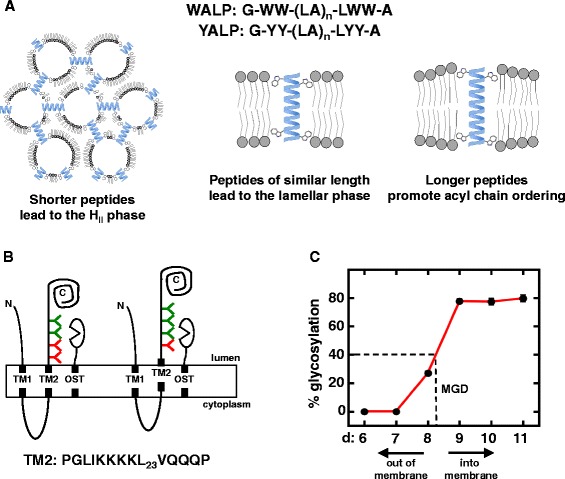
Schematic summary of the evidence supporting the affinity of amphipathic aromatic residues for polar/hydrophobic interfaces. **a** Mixing peptides consisting of a poly-Ala-Leu core of different lengths flanked by Trp residues (WALP peptides) with synthetic bilayers of different thicknesses demonstrates the affinity of the Trp residues for the polar/hydrophobic interfaces [14, 15]. When the distance between the Trp residues was sufficiently shorter than the distance between the polar/hydrophobic interfaces, the lipids adopted an inverted hexagonal (H_II_) phase to accommodate interactions between the Trp residues and the interfacial regions (*left panel*). When the distance between the flanking aromatic residues was increased so that it matched the distance between the interfacial regions, lamellar phases were observed (*center panel*). As the distance between Trp residues was increased, the acyl chains became more ordered suggesting a slight expansion of the bilayer to accommodate these “larger” peptides (*right panel*). **b** Glycosylation-mapping analysis employs a Lep model protein with two TM helices [18]. Segments to be analyzed are inserted at TM2 and a glycosylation-accepting site is positioned between 6 and 11 residues away from the lumenal boundary of TM2. Glycosylation-accepting sites shown in green are distal enough to become glycosylated while those in red are not. Proximity of an accepting site to the active site of oligosaccharyltransferase (OST) correlated with the extent of glycosylation. Therefore, repositioning of TM2 will change these relative positions and hence the extent of glycosylation. **c** Calculation of minimum glycosylation distance (MGD). MGD simply indicates the number of residues required for half-maximal glycosylation. Repositioning of TM2 into the membrane will increase MGD, while outward displacements of TM2 result in a reduction of MGD. Previous changes in MGD have demonstrated that moving Trp residues about the lumenal end of TM2 resulted in bidirectional, i.e.*,* into and out of the membrane, displacement of the TM helix [19]

Aromatic tuning was conceived based on studies with peptides that possess an aliphatic core of Ala-Leu repeats flanked by Trp (WALP) or Tyr (YALP) residues (Fig. 2a). When these WALP [14] and YALP [15] peptides were mixed with various synthetic bilayers of an appropriate thickness they were shown to adopt a transmembrane α-helical confirmation based on circular dichroism (CD), ^2^H NMR and ^31^P NMR spectroscopy. Initially, hydrophobic mismatch, or differences between the length of the aliphatic core of these peptides and the thickness of the hydrophobic bilayer core was considered to be the crucial determinant in how α-helical peptides interact with the surrounding lipidic environment [14]. Subsequently, it was shown that the contribution of interfacial anchoring, i.e.*,* interactions between Trp residues flanking the aliphatic core and the polar/hydrophobic interfaces near the boundaries of a lipid bilayer, can dominate over the effects introduced by hydrophobic mismatch alone [16]. Although this was only explicitly demonstrated for Trp residues, it was proposed that Tyr residues would also facilitate interfacial anchoring due to possessing similar physiochemical properties [16]. This elegant series of biophysical experiments is summarized in Fig. 2a. Addition of WALP or YALP peptides possessing a shorter distance between their flanking aromatic residues than the thickness of the hydrophobic core between the interfacial regions of a synthetic bilayer to which they are added results in induction of inverted hexagonal (H_II_) phases that can be detected by ^31^P NMR and sucrose density centrifugation [14, 16]. Conversely, when the distance between the aromatic residues was larger than the thickness of the hydrophobic core, the acyl chains within the bilayer became more ordered as determined by ^2^H NMR, demonstrating that the membrane is slightly expanding to accommodate these “longer” peptides [15, 17]. In essence, these experiments demonstrate that amphipathic aromatic residues, namely Trp and Tyr, possess affinity for the interfacial regions where the polar phospholipid headgroups attach to the hydrophobic acyl chains.

From a biochemical perspective, another methodology, known as glycosylation mapping, also demonstrated the contribution of aromatic residues to positioning α-helices within a lipid bilayer [18]. Glycosylation mapping utilizes the lumenally positioned endoplasmic reticulum enzyme oligosaccharyltransferase (OST) to add a glycan to the Asn residue in Asn-Xaa-(Ser/Thr) glycosylation acceptor sites. In this manner, OST acts as a molecular ruler because each acceptor site will be glycosylated to an extent that correlates with the distance between the active site of OST and the acceptor site. In Fig. 2b, the red acceptor sites are not far enough from the lumenal membrane to become glycosylated, whereas the green sites are distal enough to become glycosylated. From this information, minimum glycosylation distance (MGD), or the distance required to attain half-maximal glycosylation, can be determined as schematically shown in Fig. 2c. These MGDs can then be compared between segments to determine whether any helix repositioning occurred. Upon comparison, a decrease in MGD suggests that lumenal boundary of TM2 has been displaced into the lumen, while an increase in MGD demonstrates that the TM2 boundary has been repositioned into the membrane (Fig. 2c). A series of TM segments that are identical except for the position of the flanking aromatic residues at one end can be assessed for changes in MGD that can be assigned to the repositioning ability of those aromatic residues.

Changes in MGD have been reported when Trp residues are moved throughout the C-terminal half of a poly-Leu TM segment [19], thereby demonstrating that bidirectional repositioning of the TM segment is due to the affinity of Trp residues for the polar/hydrophobic interfaces (Fig. 2b). In a slightly modified version of this assay that considers all possible positions within the TM helix, Tyr residues where shown to possess character similar to Trp residues [20]. Thus, before aromatic tuning was conceived, previously existing biophysical and biochemical evidence suggested that Trp and Tyr residues possess affinity for the polar/hydrophobic regions located within membranes and suggested that moving them within an intact membrane-spanning protein could reposition individual TM helices.

### Aromatic residues at the ends of Tar TM2 govern its position in the membrane

**Fig. 3:**
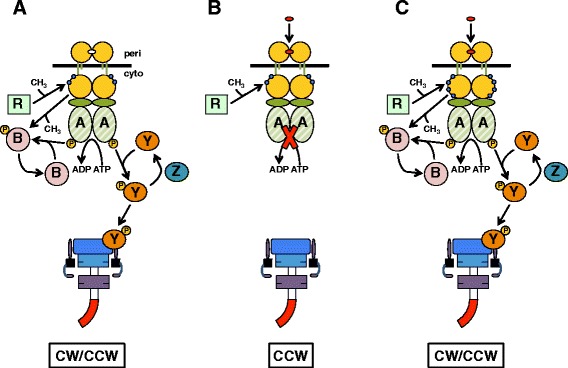
The chemotactic circuit underlying control of flagellar rotation. **a** In the absence of chemoeffectors, baseline CheA activity maintains phsopho-CheY levels that produce the three-dimensional random walk underlying canonical bacterial chemotaxis. **b** Binding of attractant (red oval in the periplasm; peri) to the chemoreceptor, i.e.*,* aspartate to Tar, abolishes CheA activity, thereby decreasing intracellular phospho-CheY levels. This also results in reduced methylesterase activity due to reduced CheB-P levels. Transmembrane communication (across the black line) is believed to occur via a piston-type displacement of TM2 toward the cytoplasm (cyto; center panel). **c** Adaptive methylation (blue dots in the cytoplasm) due to reduced CheB-P levels, restores the ability of the chemoreceptor to stimulate CheA activity when it is occupied by an attractant ligand [23]. In summary, this circuit was an excellent initial target for aromatic tuning because binding of attractant leads to displacement of TM2 toward the cytoplasm, reduced CheA kinase activity and increased levels of covalent modification. Conversely, displacements of TM2 toward the periplasm are consistent with increased CheA activity and reduced levels of covalent modification. In addition, signal output from Tar that is biased beyond the compensatory extent of methylation can be detected by monitoring rotation of individual flagella

With the aforementioned biophysical and biochemical evidence suggesting that moving flanking aromatic residues could reposition a TM helix within an intact protein, it was necessary to select an initial bacterial membrane-spanning receptor in which to examine the role of these residues in governing signal output. The aspartate chemoreceptor of *E. coli* (Tar) was chosen because it governs a well-characterized downstream signaling pathway. It is important to note that Tar is not a canonical sensor histidine kinase (SHK), requires CheW and CheA to form functional intracellular signaling complexes, and controls flagellar rotation rather than gene transcription (Fig. 3a) [21–25]. When aromatic tuning of Tar was originally undertaken, the mechanistic models for TM signaling were based upon piston-type displacements of the second transmembrane helix (TM2) [26–31]. This model proposes that displacement of TM2 toward the cytoplasm occurs upon binding of cognate ligand, i.e.*,* aspartate, to the periplasmic domain (Fig. 3b). Binding of aspartate results in squelched CheA kinase activity, which, in turn, reduces the intracellular level of phospho-CheY that promotes clockwise (CW) flagellar rotation (Fig. 3b). Phospho-CheB, also phosphorylated by CheA, is the active form of the methylesterase and is also found in reduced intracellular levels upon binding of aspartate to Tar. This results in an increase in the extent of methylation within the cytoplasmic domain of Tar, which allosterically counteracts the effect of aspartate binding and leads to restoration a normal flagellar rotational bias (Fig. 3c). One advantage to initially employing this signaling circuit was that each of these activities could be independently assessed to determine whether changes in Tar signal output have occurred. For example, it is possible to monitor CheA kinase activity *in vitro* without the complicating effects of covalent methylation [22, 26, 27]. Likewise, the extent of methylation can be examined *in vitro* in the absence of CheA kinase activity [30]. Finally, flagellar rotation can be examined *in vivo*, which will allow for determination of when signal output from Tar is biased beyond the compensatory range of methylation [26, 27].

Armed with these outputs to monitor changes in Tar signaling, initial arginyl- and cysteinyl-scanning mutagenesis of Tar TM2 from *Salmonella enteritica* serovar Typhimurium was performed and resulted in several mutations that highlighted the importance of aromatic residues in governing helix position within the cytoplasmic membrane [30]. Large changes in signal output consistent with a displacement of TM2 toward the periplasm, similar to those observed in the *apo* conformation of Tar, namely increased CheA kinase activity and decreased methylation, were observed when either Phe-189 or Trp-192 was replaced with an Arg residue at the periplasmic end of TM2. This was also seen when Phe-189 was replaced with a Cys residue. Conversely, reduced kinase activity and increased levels of covalent methylation, both consistent with the aspartate-bound confirmation of Tar, were observed when Trp-209 was replaced with Arg [30]. Independently, the importance of Trp-192 and Trp-209 in maintaining baseline signal output from *E. coli* Tar was demonstrated when they were substituted for alanyl residues [27]. In the case of the Tar W192A variant, the receptor was undermethylated and exhibited higher signal output, consistent with *apo* state and displacement of TM2 toward the periplasm. In contrast, the W209A mutant was overmethylated and possessed reduced signal output, suggesting a displacement of TM2 toward the cytoplasm as proposed to occur within the aspartate-bound confirmation. In summary, these results demonstrate that aromatic residues within TM2 of Tar are critical for maintaining normal signal output and suggest that they govern the baseline position of the helix within the membrane to an extent that could be employed to manipulate Tar signal output.

### Incremental tuning of Tar signal output

**Fig. 4:**
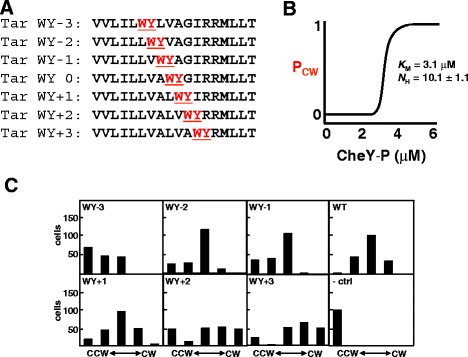
Incremental tuning of signal output from the aspartate chemoreceptor of *E. coli* (Tar). **a** At the C-terminal end of Tar TM2, a Trp-Tyr (*red*) was moved about its original position at the cytoplasmic polar/hydrophobic interface [26]. **b** The intracellular level of CheY-P governs the probability of clockwise flagellar rotation (P_CW_). Previous results demonstrate that increased intracellular CheY-P levels lead to an enhanced probability of CW rotation. In these experiments, a sharp transition was observed, i.e.*,* a Hill coefficient of more than 10 [65]. **c** Rotation of a single flagellum from roughly 200 independent *E. coli* cells expressing one of the aromatically tuned variants were analyzed and classified into one of five categories (from left to right): rotating exclusively CCW, rotating primarily CCW with occasional reversals, rapidly switching between both rotational directions (CW/CCW), rotating primarily CW with occasional reversals and those rotating exclusively CW. As Tar signal output increases, the number of cells in each category shifts from CCW toward CW rotational bias. In summary, the lowest overall signal output was observed from cells expressing the WY-3 variant, while the greatest was observed from cells expressing the WY + 2 or WY + 3 variants. Therefore, in the case of Tar, the absolute vertical position of the aromatic residues correlates with signal output [26]

Based on the contributions of the individual aromatic residues to maintenance of signal output and the biophysical and biochemical data described above, it was originally hypothesized that aromatic tuning would displace TM2 of Tar within the membrane in a step-wise manner [26]. To examine this hypothesis, a series of Tar receptors was created in which the Trp-Tyr tandem found at the cytoplasmic end of TM2 was moved up to three residue steps in either direction (Fig. 4a).

Previous results show that the intracellular concentration of CheY-P governs the clockwise flagellar (CW) rotational bias (Fig. 4b). Therefore, Tar signal output could be estimated from the aromatically tuned variants by monitoring flagellar rotation. To accomplish this, individual flagella from single *E. coli* cells were monitored for 20 s and classified into one of five categories that represent increasing levels of intracellular CheY-P: those rotating exclusively CCW, flagella rotating mostly CCW with occasional switching to CW rotation, flagella rapidly switching between CCW and CW with no inherent bias, flagella rotating mostly CW with occasional switching to CCW rotation, and finally those that rotate exclusively CW [26]. When flagella from cells expressing the aromatically tuned variants described in Fig. 4a were analyzed, a clear shift from CCW to CW rotational bias was observed as the aromatic residues were moved from WY-3 toward WY + 3 (Fig. 4c). This indicates that the signal output from the minus-series of tuned Tar variants (i.e.*,* WY-3 to WY-1) was so far biased toward reduced signal output that the compensatory action of covalent methylation failed to completely overcome this signaling bias. Conversely, the WY + 2 and WY + 3 variants were biased toward increased signal output to the extent that methylation could also not compensate. As expected, in the absence of receptor (− ctrl), only CCW rotation was observed (Fig. 4c). It is also important to note that, as predicted, overmethylation of Tar was observed for the minus-series (WY-3 to WY-1) of receptors, while undermethylation was seen for the plus-series (WY + 1 to WY + 3) of aromatically tuned variants [26]. When taken together, these results demonstrate that signal output from Tar is modulated in an incremental manner when the aromatic residues were moved at the cytoplasmic end of TM2.

While these results served as an interesting foray into aromatic tuning, they do not empirically demonstrate that moving the aromatic residues at the cytoplasmic end of TM2 results in a physical repositioning of the helix. Therefore, two subsequent studies, one computational and one biochemical, were performed to assess repositioning of Tar TM2 upon moving the Trp-Tyr tandem. Coarse-grained molecular dynamics (CG-MD) simulations that were employed to examine the ability of aromatic tuning to displace Tar TM2 in the presence of an explicit membrane and solvent demonstrated that moving the Trp-Tyr residue was sufficient to induce small TM2 displacements of up to 1.5 Å [32]. In addition, the glycosylation-mapping assay described above (Fig. 2b and c) was performed in order to determine whether moving the Trp-Tyr pair at the end of TM2 would yield analogous results in a biological membrane. Assuming that the region in Lep that contains the glycosylation-accepting site is in an extended conformation, a shift in MGD of 0.5 residues as seen for the Tar constructs corresponds to a shift in the positioning of the TM2 helix of 1.6-1.7 Å [33], in close agreement with the CG-MD results [32]. Furthermore, within both the CG-MD simulations and MGD analysis, similar patterns of displacement were observed. A grouping of the minus-series of receptors with similar displacements toward the cytoplasm (WY-3 through WY-1), a baseline position for the wild-type (WY 0), two receptors that are slightly displaced toward the periplasm (WY + 1 and WY + 2) and a larger shift toward the periplasm for the WY + 3 variant was observed with both techniques. Therefore, this combination of *in vivo*, *in vitro* and *in silico* results demonstrate that repositioning the Trp-Tyr tandem at the cytoplasmic end is sufficient to generate a physical displacement of TM2 and the resulting modulation of Tar signal output in an incremental manner.

### Non-incremental tuning of EnvZ signal output

In order to determine whether aromatic tuning would work within a canonical SHK, its effectiveness was examined within the major *E. coli* osmosensor (EnvZ), where a rotation of TM2 was initially proposed as the mechanism of transmembrane communication [34–38]. More recently, a regulated unfolding [39] model has been suggested, in which SHKs are modular proteins composed of individually folding domains that each contribute a distinct functionality. Regulated unfolding suggests that the effector domain within an SHK is maintained in an inactive conformation by a rigid connection between the stimulus perception and effector domains. Upon perception of stimulus, this connection disengages allowing the effector domain to adopt an active conformation [39]. This region connecting the TM to the cytoplasmic domain in bacterial receptors is colloquially referred to as a “control cable” because its residue composition governs coupling of signal transduction between adjacent domains [26, 27, 40–47].

**Fig. 5:**
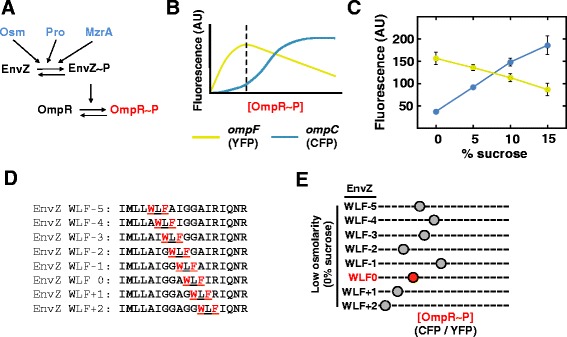
Non-incremental tuning of signal output from the major osmosensor of *E. coli* (EnvZ). **a** EnvZ is bifunctional and possesses both kinase and phosphatase activity. The ratio of these activities is modulated by several factors including the presence of extracellular osmolarity [66, 67], procaine [68] or MzrA [69, 70]. OmpR serves as the cognate RR of EnvZ and the intracellular level of phosphorylated OmpR (OmpR-P) in governed by EnvZ activity. **b** OmpR-P levels control transcription of *ompF* and *ompC,* which can be monitored by employing an *E. coli* strain that contains a transcriptional fusion of *yfp* to *ompF* (*yellow*) and of *cfp* to *ompC* (*blue*) [7]. Intracellular levels of OmpR-P (*red*) can thus be estimated by monitoring the CFP/YFP ratio. **c** Experimental results demonstrating that increasing sucrose levels in the growth medium resulted in increasing levels of CFP, decreasing levels of YFP and an increase in the CFP/YFP ratio. The black dashed line in Fig. 5B represents signal output from this strain upon growth in medium with no additional sucrose. **d** When aromatic tuning was performed in EnvZ, a Trp-Leu-Phe triplet (*red*) was repositioned within the C-terminal region of TM2. **e** The gray-filled circles on the dashed lines indicate the estimated OmpR-P levels in cells expressing one of the aromatically tuned variants. Aromatic tuning in EnvZ resulted in a pattern of signal output that did not correlate with the absolute vertical position of the aromatic residues, as was the case with Tar, but rather appeared approximately helical in distribution suggesting that the surface of TM2 that the residues were located upon was of greater importance [33]. However, the key outcome is that aromatic tuning was still successful with respect to modulating EnvZ signal output in a stimulus-independent manner

EnvZ is a canonical SHK that responds to changes in the extracellular osmolarity of inner-membrane impermeable compounds by modulating the intracellular level of phosphorylated OmpR (Fig. 5a) [48–51]. In a laboratory environment, EnvZ signal output is modulated by simply adding sucrose to the bacterial growth medium. Subsequently, phospho-OmpR regulates the transcription of a number of genes, including those encoding two outer membrane porins, OmpF and OmpC. At low intracellular levels of phospho-OmpR (OmpR-P), transcription of *ompF* is upregulated, whereas at higher levels of OmpR-P, transcription of *ompF* is repressed and transcription of *ompC* is activated. This results in a predominance of OmpF at low osmolarity and OmpC at higher osmolarities (Fig. 5b) [52–54]. The easily controllable nature of the input stimulus, i.e.*,* addition of sucrose, and the well-characterized transcriptional output made the EnvZ/OmpR osmosensing circuit an ideal choice for examining aromatic tuning within an SHK.

To analyze steady-state signal output from EnvZ/OmpR osmosensing circuits containing aromatically tuned receptors, a two-color fluorescent reporter strain was employed. This *E. coli* strain possesses transcriptional fusions of *cfp* to *ompC* and of *yfp* to *ompF* within its chromosome (Fig. 5b) [55]. Quantifying the ratio of CFP to YFP fluorescence provides a rapid and sensitive measure of the ratio of *ompC* to *ompF* transcription, which estimates the intracellular level of phosphoylated OmpR. Therefore, the ratio of CFP to YFP can be employed to estimate signal output from the aromatically tuned EnvZ variants. To confirm this, we previously demonstrated that the transcription of CFP and YFP behaved as expected upon addition of sucrose to the growth medium (Fig. 5c) [7].

Due to the proposal of non-piston signaling mechanisms, it was difficult to predict what pattern of signal outputs would be observed upon aromatic tuning of an SHK. In the case of EnvZ, a Trp-Leu-Phe triplet was moved from five residues into the membrane (WLF-5) through two residues out of the membrane (WLF + 2) in single residue steps (Fig. 5d). In this case, the surface of TM2 that the aromatic residues reside upon was found to be the major determinant of EnvZ signal output rather than their absolute vertical position as was the case with Tar (Fig. 5E) [7]. These results clearly demonstrated that even though a different mechanism of TM communication has been proposed, aromatic tuning remained successful in modulating signal output. Similar results were obtained with a Trp-Tyr-Ala triplet that is more similar to the Trp-Tyr tandem employed during tuning of Tar suggesting that in the case of EnvZ, the composition of the residues that are moved is not a major determinant in the pattern of signal output [7]. However, additional experimentation is required to support the concept that disturbance of a particular helical surface is the cause of changes in EnvZ signal output. It should also be noted that these different patterns of signal output modulation are not surprising given that the concept of various signaling mechanisms being employed by different subclasses of bacterial receptors has been recently put forward [56–58]. Therefore, regardless of whichever signaling mechanism is employed by a receptor, aromatic tuning may be successful in modulating signal output.

Glycosylation mapping experiments with TM2 of EnvZ demonstrated that moving the Trp-Leu-Phe triplet resulted in almost no change in MGD. An exception was observed for WLF-3, which is most likely a specific case where the positive charge within the side-chain of Arg-182 would not be able to interact with the headgroups of the membrane any longer due to it being displaced very far out of the membrane. Thus, in the case of most aromatically tuned EnvZ segments, changes in MGD are very small and not steadily increasing in contrast to what was observed with the aromatically tuned Tar TM2 segments [33]. Initial CG-MD analysis with these aromatically tuned EnvZ segments also does not support incremental displacement (B. Hall, personal communication).

### Employment of aromatic tuning within a wide variety of membrane-spanning receptors

Aromatic tuning was successful in modulating signal output from both Tar and EnvZ, however, a difference in the pattern of signal outputs was observed [7, 26]. This pattern of signal outputs shows that even though aromatic tuning did not displace the TM2 helix of EnvZ [33], it was still effective in modulating signal output from the full-length receptor in vivo. Aromatic tuning has also been successfully employed in two different Tar-EnvZ chimeric receptors (Lehning and Draheim, unpublished observations), when a Trp-Tyr tandem was used, and within a NarX-Tar chimera (Reinhard and Draheim, unpublished observations), when a single Trp residue was moved.

Published sequence alignments demonstrate that aromatic residues are often found at the cytoplasmic end of the final transmembrane helix within bacterial membrane-spanning receptors [27, 59] suggesting that aromatic tuning could be useful for research groups working with other two-component circuits. It is important to note that the majority of aromatically tuned Tar, EnvZ and chimeric receptor variants retain the ability to respond to stimulus suggesting that their signal output is biased but not locked in either a stimulus-deprived or a stimulus-saturated conformation. In this regard, aromatic tuning is advantageous compared to deletion of entire SHKs [60] or substitution of the conserved His residue involved in autophosphorylation and phosphotransfer because these methods may result in complete loss of kinase or phosphatase activity, which has been shown to result in non-physiological cross-talk between various two-component signaling pathways within a cell [61, 62]. Thus, by apparently biasing, rather than abolishing activity and stimulus-perception, aromatic tuning represents a significant improvement over previous attempts at stimulus-independent modulation of signal output. Even though these initial attempts have been successful, we have only observed aromatic tuning in membrane-spanning receptors that possess two TM helices. However, we feel strongly that in the case of SHKs containing only two TM helices, that aromatic tuning will facilitate stimulus-independent modulation in the majority of cases. Furthermore, we are currently expanding our range of targets to include SHKs that possess more than two TMs such as AgrC from *Staphylococcus aureus* that has recently been shown to possess seven TMs [63].

In addition to rectifying issues with signal output or stimulus-perception, we believe that aromatic tuning has other potential uses. For example, the input stimulus has been identified for only a small fraction of SHKs, thereby making it challenging to investigate downstream signaling pathways without extensive mutagenesis and subsequent screening for changes in phenotypic output. Aromatic tuning could be employed to circumvent these limitations and allow manipulation of signal output from a specifically targeted SHK. One potential use would be the rapid assignment of downstream physiological and developmental processes to particular SHKs. Each SHK within an organism could be independently subjected to aromatic tuning and subsequent monitoring for the phenotype of interest. If the appearance of the phenotype correlated with aromatic tuning of a particular SHK, this would suggest that the desired phenotype is governed by the aromatically tuned receptor. In addition to assignment of phenotypes to specific SHKs, aromatic tuning could be employed to facilitate induction of medically relevant bacterial phenotypes in the absence of complex host-pathogen interactions, thus reducing the burden of complex interkingdom laboratory model systems. Finally, aromatic tuning of individual SHKs could be coupled with pre-existing transcriptional reporter libraries [64] rather than observable phenotypes, to rapidly unravel the signaling pathways governed by each SHK. This would facilitate rapid and cost-effective organism-level signal pathway mapping.

## Conclusion

The aromatic residues described here are found at the cytoplasmic boundary of the final TM helix within many bacterial membrane-spanning receptors, suggesting that aromatic tuning may useful in a wide-range of applications involving synthetic microbiology. Here, we provide a brief synopsis of the data supporting aromatic tuning, its initial successes and the advantages of this method over those previously employed for modulating TCS signal output. We have shown that aromatic tuning works within two well-characterized systems and hope that it is presented in sufficient detail to provide other research groups with the opportunity to employ the methodology within their TCSs of interest.

## Abbreviations

TCS: Two-component signaling circuit
ORF: Open reading frame
SHK: Sensor histidine kinase
RR: Response regulator
WALP: Trp-flanked peptides with a poly-Ala-Leu core
YALP: Tyr-flanked peptides with a poly-Ala-Leu core
CD: Circular dichroism spectroscopy
NMR: Nuclear magnetic resonance spectroscopy
OST: Oligosaccharyltransferase
MGD: Minimum glycosylation distance
TM: Transmembrane
TM2: Second transmembrane helix
CCW: Counterclockwise
CW: Clockwise
CG-MD: Course-grained molecular dynamics simulations
CA: Domain responsible for catalytic ATPase activity
DHp: Domain responsible for dimerization and histidylphosphotransfer

## References

[1] Stock AM, Robinson VL, Goudreau PN (2000). Two-component signal transduction. Annu Rev Biochem.

[2] Ji G, Beavis R, Novick RP (1997). Bacterial interference caused by autoinducing peptide variants. Science.

[3] Shin D, Lee EJ, Huang H, Groisman EA (2006). A positive feedback loop promotes transcription surge that jump-starts Salmonella virulence circuit. Science.

[4] Puthiyaveetil S, Kavanagh TA, Cain P, Sullivan JA, Newell CA, Gray JC (2008). The ancestral symbiont sensor kinase CSK links photosynthesis with gene expression in chloroplasts. Proc Natl Acad Sci U S A.

[5] David M, Daveran ML, Batut J, Dedieu A, Domergue O, Ghai J (1988). Cascade regulation of nif gene expression in Rhizobium meliloti. Cell.

[6] Ulrich LE, Zhulin IB (2010). The MiST2 database: a comprehensive genomics resource on microbial signal transduction. Nucleic Acids Res.

[7] Norholm MH, von Heijne G, Draheim RR (2015). Forcing the Issue: Aromatic Tuning Facilitates Stimulus-Independent Modulation of a Two-Component Signaling Circuit. ACS Synth Biol.

[8] Wang B, Barahona M, Buck M, Schumacher J (2013). Rewiring cell signalling through chimaeric regulatory protein engineering. Biochem Soc Trans.

[9] Stanton BC, Siciliano V, Ghodasara A, Wroblewska L, Clancy K, Trefzer AC (2014). Systematic transfer of prokaryotic sensors and circuits to Mammalian cells. ACS Synth Biol.

[10] Zhu Y, Inouye M (2003). Analysis of the role of the EnvZ linker region in signal transduction using a chimeric Tar/EnvZ receptor protein, Tez1. J Biol Chem.

[11] Jung K, Fried L, Behr S, Heermann R (2012). Histidine kinases and response regulators in networks. Curr Opin Microbiol.

[12] Mascher T, Helmann JD, Unden G (2006). Stimulus perception in bacterial signal-transducing histidine kinases. Microbiol Mol Biol R.

[13] Russo FD, Silhavy TJ (1993). The essential tension: opposed reactions in bacterial two-component regulatory systems. Trends Microbiol.

[14] Killian JA, Salemink I, de Planque MR, Lindblom G, Koeppe RE, Greathouse DV (1996). Induction of nonbilayer structures in diacylphosphatidylcholine model membranes by transmembrane alpha-helical peptides: importance of hydrophobic mismatch and proposed role of tryptophans. Biochemistry.

[15] de Planque MR, Boots JW, Rijkers DT, Liskamp RM, Greathouse DV, Killian JA (2002). The effects of hydrophobic mismatch between phosphatidylcholine bilayers and transmembrane alpha-helical peptides depend on the nature of interfacially exposed aromatic and charged residues. Biochemistry.

[16] de Planque MR, Bonev BB, Demmers JA, Greathouse DV, Koeppe RE, Separovic F (2003). Interfacial anchor properties of tryptophan residues in transmembrane peptides can dominate over hydrophobic matching effects in peptide-lipid interactions. Biochemistry.

[17] de Planque MR, Greathouse DV, Koeppe RE, Schafer H, Marsh D, Killian JA (1998). Influence of lipid/peptide hydrophobic mismatch on the thickness of diacylphosphatidylcholine bilayers. A 2H NMR and ESR study using designed transmembrane alpha-helical peptides and gramicidin A. Biochemistry.

[18] Nilsson I, Saaf A, Whitley P, Gafvelin G, Waller C, von Heijne G (1998). Proline-induced disruption of a transmembrane alpha-helix in its natural environment. J Mol Biol.

[19] Braun P, von Heijne G (1999). The aromatic residues Trp and Phe have different effects on the positioning of a transmembrane helix in the microsomal membrane. Biochemistry.

[20] Hessa T, Meindl-Beinker NM, Bernsel A, Kim H, Sato Y, Lerch-Bader M (2007). Molecular code for transmembrane-helix recognition by the Sec61 translocon. Nature.

[21] Welch M, Oosawa K, Aizawa S, Eisenbach M (1993). Phosphorylation-dependent binding of a signal molecule to the flagellar switch of bacteria. Proc Natl Acad Sci U S A.

[22] Lai RZ, Manson JM, Bormans AF, Draheim RR, Nguyen NT, Manson MD (2005). Cooperative signaling among bacterial chemoreceptors. Biochemistry.

[23] Draheim RR (2007). The role of protein-membrane interactions in modulation of signaling by bacterial chemoreceptors.

[24] Ravid S, Matsumura P, Eisenbach M (1986). Restoration of flagellar clockwise rotation in bacterial envelopes by insertion of the chemotaxis protein CheY. Proc Natl Acad Sci U S A.

[25] Hess JF, Oosawa K, Kaplan N, Simon MI (1988). Phosphorylation of three proteins in the signaling pathway of bacterial chemotaxis. Cell.

[26] Draheim RR, Bormans AF, Lai RZ, Manson MD (2006). Tuning a bacterial chemoreceptor with protein-membrane interactions. Biochemistry.

[27] Draheim RR, Bormans AF, Lai RZ, Manson MD (2005). Tryptophan residues flanking the second transmembrane helix (TM2) set the signaling state of the Tar chemoreceptor. Biochemistry.

[28] Falke JJ, Hazelbauer GL (2001). Transmembrane signaling in bacterial chemoreceptors. Trends Biochem Sci.

[29] Falke JJ, Erbse AH (2009). The piston rises again. Structure.

[30] Miller AS, Falke JJ (2004). Side chains at the membrane-water interface modulate the signaling state of a transmembrane receptor. Biochemistry.

[31] Isaac B, Gallagher GJ, Balazs YS, Thompson LK (2002). Site-directed rotational resonance solid-state NMR distance measurements probe structure and mechanism in the transmembrane domain of the serine bacterial chemoreceptor. Biochemistry.

[32] Hall BA, Armitage JP, Sansom MSP (2011). Transmembrane Helix Dynamics of Bacterial Chemoreceptors Supports a Piston Model of Signalling. PLoS Comput Biol.

[33] Botelho SC, Enquist K, von Heijne G, Draheim RR (2015). Differential repositioning of the second transmembrane helices from E. coli Tar and EnvZ upon moving the flanking aromatic residues. Biochim Biophys Acta.

[34] Ferris HU, Dunin-Horkawicz S, Mondejar LG, Hulko M, Hantke K, Martin J (2011). The mechanisms of HAMP-mediated signaling in transmembrane receptors. Structure.

[35] Ferris HU, Dunin-Horkawicz S, Hornig N, Hulko M, Martin J, Schultz JE (2012). Mechanism of regulation of receptor histidine kinases. Structure.

[36] Ferris HU, Zeth K, Hulko M, Dunin-Horkawicz S, Lupas AN (2014). Axial helix rotation as a mechanism for signal regulation inferred from the crystallographic analysis of the E. coli serine chemoreceptor. J Struct Biol.

[37] Hulko M, Berndt F, Gruber M, Linder JU, Truffault V, Schultz A (2006). The HAMP domain structure implies helix rotation in transmembrane signaling. Cell.

[38] Inouye M (2006). Signaling by transmembrane proteins shifts gears. Cell.

[39] Schultz JE, Natarajan J (2013). Regulated unfolding: a basic principle of intraprotein signaling in modular proteins. Trends Biochem Sci.

[40] Parkinson JS (2010). Signaling mechanisms of HAMP domains in chemoreceptors and sensor kinases. Annu Rev Microbiol.

[41] Zhou Q, Ames P, Parkinson JS (2011). Biphasic Control Logic of HAMP Domain Signaling in the Escherichia coli Serine Chemoreceptor. Mol Microbiol.

[42] Kitanovic S, Ames P, Parkinson JS (2011). Mutational analysis of the control cable that mediates transmembrane signaling in the Escherichia coli serine chemoreceptor. J Bacteriol.

[43] Park H, Im W, Seok C (2011). Transmembrane signaling of chemotaxis receptor tar: insights from molecular dynamics simulation studies. Biophys J.

[44] Wright GA, Crowder RL, Draheim RR, Manson MD (2011). Mutational analysis of the transmembrane helix 2-HAMP domain connection in the Escherichia coli aspartate chemoreceptor tar. J Bacteriol.

[45] Adase CA, Draheim RR, Rueda G, Desai R, Manson MD (2013). Residues at the cytoplasmic end of transmembrane helix 2 determine the signal output of the TarEc chemoreceptor. Biochemistry.

[46] Adase CA, Draheim RR, Manson MD (2012). The residue composition of the aromatic anchor of the second transmembrane helix determines the signaling properties of the aspartate/maltose chemoreceptor Tar of Escherichia coli. Biochemistry.

[47] Zhou Q, Ames P, Parkinson JS (2009). Mutational analyses of HAMP helices suggest a dynamic bundle model of input–output signalling in chemoreceptors. Mol Microbiol.

[48] Egger LA, Park H, Inouye M (1997). Signal transduction via the histidyl-aspartyl phosphorelay. Genes Cells.

[49] Forst SA, Roberts DL (1994). Signal transduction by the EnvZ-OmpR phosphotransfer system in bacteria. Res Microbiol.

[50] Hoch JA, Silhavy TJ (1995). Two-Component Signal Transduction.

[51] Mizuno T (1998). His-Asp phosphotransfer signal transduction. J Biochem.

[52] Forst S, Delgado J, Rampersaud A, Inouye M (1990). In vivo phosphorylation of OmpR, the transcription activator of the ompF and ompC genes in Escherichia coli. J Bacteriol.

[53] Lan CY, Igo MM (1998). Differential expression of the OmpF and OmpC porin proteins in Escherichia coli K-12 depends upon the level of active OmpR. J Bacteriol.

[54] Russo FD, Silhavy TJ (1991). EnvZ controls the concentration of phosphorylated OmpR to mediate osmoregulation of the porin genes. J Mol Biol.

[55] Batchelor E, Silhavy TJ, Goulian M (2004). Continuous control in bacterial regulatory circuits. J Bacteriol.

[56] Falke JJ (2014). Piston versus Scissors: Chemotaxis Receptors versus Sensor His-Kinase Receptors in Two-Component Signaling Pathways. Structure.

[57] Unnerstale S, Maler L, Draheim RR (2011). Structural characterization of AS1-membrane interactions from a subset of HAMP domains. Biochim Biophys Acta.

[58] Molnar KS, Bonomi M, Pellarin R, Clinthorne GD, Gonzalez G, Goldberg SD (2014). Cys-Scanning Disulfide Crosslinking and Bayesian Modeling Probe the Transmembrane Signaling Mechanism of the Histidine Kinase. PhoQ, Structure.

[59] Boldog T, Hazelbauer GL (2004). Accessibility of introduced cysteines in chemoreceptor transmembrane helices reveals boundaries interior to bracketing charged residues. Protein Sci.

[60] Zhou L, Lei XH, Bochner BR, Wanner BL (2003). Phenotype microarray analysis of Escherichia coli K-12 mutants with deletions of all two-component systems. J Bacteriol.

[61] Groban ES, Clarke EJ, Salis HM, Miller SM, Voigt CA (2009). Kinetic buffering of cross talk between bacterial two-component sensors. J Mol Biol.

[62] Siryaporn A, Goulian M (2010). Characterizing cross-talk in vivo avoiding pitfalls and overinterpretation. Methods Enzymol.

[63] Wang L, Quan C, Xiong W, Qu X, Fan S, Hu W (2014). New insight into transmembrane topology of Staphylococcus aureus histidine kinase AgrC. Biochim Biophys Acta.

[64] Zaslaver A, Bren A, Ronen M, Itzkovitz S, Kikoin I, Shavit S (2006). A comprehensive library of fluorescent transcriptional reporters for Escherichia coli. Nat Methods.

[65] Cluzel P, Surette M, Leibler S (2000). An ultrasensitive bacterial motor revealed by monitoring signaling proteins in single cells. Science.

[66] Egger LA, Inouye M (1997). Purification and characterization of the periplasmic domain of EnvZ osmosensor in Escherichia coli. Biochem Biophys Res Commun.

[67] Forst S, Delgado J, Inouye M (1989). Phosphorylation of OmpR by the osmosensor EnvZ modulates expression of the ompF and ompC genes in Escherichia coli. Proc Natl Acad Sci U S A.

[68] Rampersaud A, Inouye M (1991). Procaine, a local anesthetic, signals through the EnvZ receptor to change the DNA binding affinity of the transcriptional activator protein OmpR. J Bacteriol.

[69] Gerken H, Charlson ES, Cicirelli EM, Kenney LJ, Misra R (2009). MzrA: a novel modulator of the EnvZ/OmpR two-component regulon. Mol Microbiol.

[70] Gerken H, Misra R (2010). MzrA-EnvZ interactions in the periplasm influence the EnvZ/OmpR two-component regulon. J Bacteriol.

